# Identification and immune characteristics of molecular subtypes related to protein glycosylation in Alzheimer’s disease

**DOI:** 10.3389/fnagi.2022.968190

**Published:** 2022-11-02

**Authors:** Zhaotian Ma, Fan Yang, Jiajia Fan, Xin Li, Yuanyuan Liu, Wei Chen, Honghao Sun, Tengfei Ma, Qiongying Wang, Yueriguli Maihaiti, Xiaoqiao Ren

**Affiliations:** ^1^School of Traditional Chinese Medicine, Beijing University of Chinese Medicine, Beijing, China; ^2^Institute of Ethnic Medicine, Beijing University of Chinese Medicine, Beijing, China; ^3^National Institute of Traditional Chinese Medicine (TCM) Constitution and Preventive Medicine, Beijing University of Chinese Medicine, Beijing, China

**Keywords:** Alzheimer’s disease, diagnostic model, immune cells, protein glycosylation-related genes, molecular subtypes

## Abstract

**Background:**

Protein glycosylation has been confirmed to be involved in the pathological mechanisms of Alzheimer’s disease (AD); however, there is still a lack of systematic analysis of the immune processes mediated by protein glycosylation-related genes (PGRGs) in AD.

**Materials and methods:**

Transcriptomic data of AD patients were obtained from the Gene Expression Omnibus database and divided into training and verification datasets. The core PGRGs of the training set were identified by weighted gene co-expression network analysis, and protein glycosylation-related subtypes in AD were identified based on k-means unsupervised clustering. Protein glycosylation scores and neuroinflammatory levels of different subtypes were compared, and functional enrichment analysis and drug prediction were performed based on the differentially expressed genes (DEGs) between the subtypes. A random forest model was used to select important DEGs as diagnostic markers between subtypes, and a line chart model was constructed and verified in other datasets. We evaluated the differences in immune cell infiltration between the subtypes through the single-sample gene set enrichment analysis, analyzed the correlation between core diagnostic markers and immune cells, and explored the expression regulation network of the core diagnostic markers.

**Results:**

Eight core PGRGs were differentially expressed between the training set and control samples. AD was divided into two subtypes with significantly different biological processes, such as vesicle-mediated transport in synapses and neuroactive ligand-receptor interactions. The high protein glycosylation subtype had a higher level of neuroinflammation. Riluzole and sulfasalazine were found to have potential clinical value in this subtype. A reliable construction line chart model was constructed based on nine diagnostic markers, and *SERPINA3* was identified as the core diagnostic marker. There were significant differences in immune cell infiltration between the two subtypes. *SERPINA3* was found to be closely related to immune cells, and the expression of *SERPINA3* in AD was found to be regulated by a competing endogenous RNA network that involves eight long non-coding RNAs and seven microRNAs.

**Conclusion:**

Protein glycosylation and its corresponding immune process play an important role in the occurrence and development of AD. Understanding the role of PGRGs in AD may provide a new potential therapeutic target for AD.

## Introduction

Alzheimer’s disease (AD) is an irreversible, progressive, polygenic, neurodegenerative disease that accounts for approximately 70% of dementia cases (Li et al., 2022; Tank et al., 2022). The onset of AD is unknown, and nerves undergo pathological changes decades before the onset of symptoms (Alzheimer’s Association, 2021). Exploring the prominent clinical features of AD, including its complex etiology and high phenotypic heterogeneity, is essential because the exact pathogenesis of AD remains to be fully elucidated, which limits the development of effective drugs (Servick, 2021; Gherardelli et al., 2022). The course of disease evolution and differences in drug sensitivity among patients are generally believed to be related to the molecular heterogeneity of the disease (Mohamed Abd-El-Halim et al., 2021). Therefore, considering that existing therapeutic drugs and regimens can only slow down the progression of AD but not prevent or reverse it, evaluating the types of AD based on specific molecular mechanisms and developing corresponding therapeutic drugs is an effective strategy for achieving accurate medical goals.

Protein glycosylation, the process by which glycosidic chains form glycosidic bonds with certain amino acid residues on proteins catalyzed by glycosyltransferases, is an important posttranslational modification that occurs in 50–70% of proteins in cells. According to the glycoside chain type, protein glycosylation modification is mainly divided into four types, of which N-linked and O-linked glycosylation are the two main modification types (Schjoldager et al., 2020). Protein glycosylation regulates the function and activity of proteins, affecting many important cellular activities, such as cell recognition, differentiation, signal transduction, and immune response. Protein glycosylation disorders affect the pathological mechanisms of AD by mediating a variety of biological processes, such as neuroinflammation and cellular signal transduction (Zhang et al., 2020). For example, tau is a microtubule-associated protein, and the total level of tau in cerebrospinal fluid reflects the degree of neuronal damage in AD (Zetterberg, 2017). Tau has shown significant N-linked glycosylation in AD brain tissue but not in the normal brain (Wang et al., 1996). The hydrolysis of N-linked glycosylated amyloid β precursor protein (APP) to amyloid β peptide is a marker of AD, and O-linked glycosylation interferes with the proteolysis of APP and affects the pathological changes related to AD (Singh et al., 2022). Additionally, protein glycosylation is closely related to immune cells and immune response in AD. Some studies have found that most of the glycoproteins with site-specific glycosylation in the serum of patients with AD are involved in immune function and induce specific inflammatory pathways and immune responses (Tena et al., 2022). Although existing studies have preliminarily revealed the relationship of protein glycosylation and protein glycosylation-related genes (PGRGs) with AD, there is still a lack of comprehensive analysis of the role of PGRGs in the occurrence and development of AD combined with immune imbalance.

In this study, we screened the key PGRGs related to AD and explored the differences in immune cell infiltration under different PGRG expression patterns. Based on a machine learning method, a diagnostic model of a high-risk PGRG subtype was established, potential therapeutic drugs were screened, and the core genes of the model were verified in other datasets. These results provide a reference for protein glycosylation as a therapeutic target for AD.

## Materials and methods

### Data source

The microarray datasets GSE5281, GSE33000, GSE185909, GSE118553, GSE122063, GSE44768, and GSE44770 were downloaded from the Gene Expression Omnibus (GEO) database.^1^ These datasets included brain samples from 1,029 AD patients and 585 non-demented elderly individuals. GSE118553, GSE44768, GSE44770, and GSE122063 were used as verification datasets (Liang et al., 2007; Narayanan et al., 2014; McKay et al., 2019). The original data were batch-corrected for further analyses. Differentially expressed genes (DEGs) were screened using the “limma” software package in R, and *P* < 0.05 and | log_2_FC| > 1 were considered statistically significant (Ritchie et al., 2015). PGRGs were extracted from the GlycoGene^2^ and HUGO Gene Nomenclature Committee^3^ databases. In addition, PGRGs with a correlation score higher than 15 were extracted from the GeneCards database.^4^ The three gene lists were combined and integrated into a protein glycosylation gene set.

### Weighted gene co-expression network analysis

The protein glycosylation gene enrichment score (GLY) of all samples was calculated by gene set variation analysis (GSVA), and weighted gene co-expression network analysis (WGCNA) was carried out with the R package “WGCNA” according to the GLY (Langfelder and Horvath, 2008; Langfelder et al., 2012). The adjacency matrix of weighted correlation coefficients was transformed into a topological overlap matrix (TOM) and corresponding dissimilarity matrix (1-TOM). Hierarchical clustering was performed, a system cluster diagram was constructed, and genes with similar expression profiles were divided into different modules. Finally, Pearson’s correlation analysis was used to assess the correlation between various phenotypes, including the GLY and the groupings, and each module. Genes in the module with the highest correlation coefficient were considered to be the most related to AD. The transcriptional regulatory network of key modules was predicted using the ChEA3 database,^5^ which includes a large number of independently published CHIP-seq datasets and integrates transcription factor co-expression data based on RNA-seq data (Fu et al., 2021).

### Classification and functional enrichment analysis of protein glycosylation-related genes-related subtypes in Alzheimer’s disease

The intersection of the key module genes obtained by the WGCNA analysis and differentially expressed PGRGs was obtained, and an unsupervised cluster analysis was performed to identify different AD subtypes. In parallel, principal component analysis (PCA) was applied to calculate the protein glycosylation levels for each case in the subtypes to generate a protein glycosylation score (Glyscore). A consensus clustering algorithm was used to evaluate the cluster numbers and robustness. The R package “ConsensusClusterPlus” implements the above steps for 1,000 iterations to guarantee the robustness of the classification (Wilkerson and Hayes, 2010). Gene set enrichment analysis (GSEA) was performed on the gene expression matrix using the “cluster profile” package, and “c2.cp.kegg.v7.0.symbols.gmt” was selected as the reference gene set. We used the “path view,” “ggplot2,” and “circlize” packages to perform Gene Ontology (GO) and Kyoto Encyclopedia of Genes and Genomes (KEGG) enrichment analyses on the DEGs (Ginestet, 2011; Luo and Brouwer, 2013; Gu et al., 2014).

### Identification of protein glycosylation-related genes-related small molecule therapeutic drugs

The Broad Institutes Connectivity Map (cMAP) database^6^ was used to identify small molecule drugs related to different PGRG subtypes in AD (Lamb et al., 2006). To identify candidate drugs, gene sets with log_2_FC > 0.585 were input into the cMAP database for enrichment analysis. The accuracy of the results was verified by molecular docking. The PubChem database^7^ was used to extract the characteristics of the small molecules and obtain their 3D structures. Subsequently, molecular docking was performed to retrieve the crystal structure information (human origin) of the predicted target protein using the PDB database,^8^ and the receptor protein was pretreated using the AutoDockTool software for dehydrogenation, hydrogenation, etc. Molecular docking of target proteins with small molecular drugs was carried out using the Discovery Studio 4.5.0 software, and then the binding ability of the two molecules was predicted. The possibility of significant binding was considered when the binding energy was negative and the absolute value was greater than 5 kcal/mol.

### Screening, expression regulation, and immune cell infiltration analysis of diagnostic markers among protein glycosylation subtypes

Random forest is a widely used method for accurately calculating the importance of each feature in a dataset. In this study, the “randomForest” package in R was used to select the key differential genes between the AD subtypes as diagnostic markers, and the “rms” package was used to construct the prognosis diagram of key differential genes (Liaw and Wiener, 2002). A calibration curve was then used to evaluate the predictive ability of the line chart model. Finally, the clinical value of the model was evaluated using decision curve analysis (DCA) and clinical impact curve (Pan et al., 2021). The selected key differential genes were verified in the verification dataset, and genes with a higher diagnostic efficiency were identified as diagnostic markers. The prediction of non-coding RNA regulation of diagnostic markers was first analyzed using the RNA22, miRWalk, miRDB, and RNAInter databases to select intersecting microRNAs (miRNAs) (Li et al., 2014). We selected miRNAs that have been reported to play a biological role in AD for further analyses. The MiRNet2.0^9^ and starBase^10^ databases were used to predict the target long non-coding RNAs (lncRNAs) of the miRNAs. Lastly, the competing endogenous RNA (ceRNA) network was established (Chang et al., 2020). The degree of immune cell infiltration in brain tissue was evaluated by the single sample GSEA (ssGSEA) algorithm, and the difference in immune cell infiltration between groups was visualized by the “ggplot2” package (Newman et al., 2015; Deng et al., 2020). Finally, Spearman correlation analysis was performed for all immune cells and diagnostic markers, and the positive or negative correlation between them was determined by the “ggstatsplot” package.

## Results

### Identification of core protein glycosylation-related genes in Alzheimer’s disease

**FIGURE 1:**
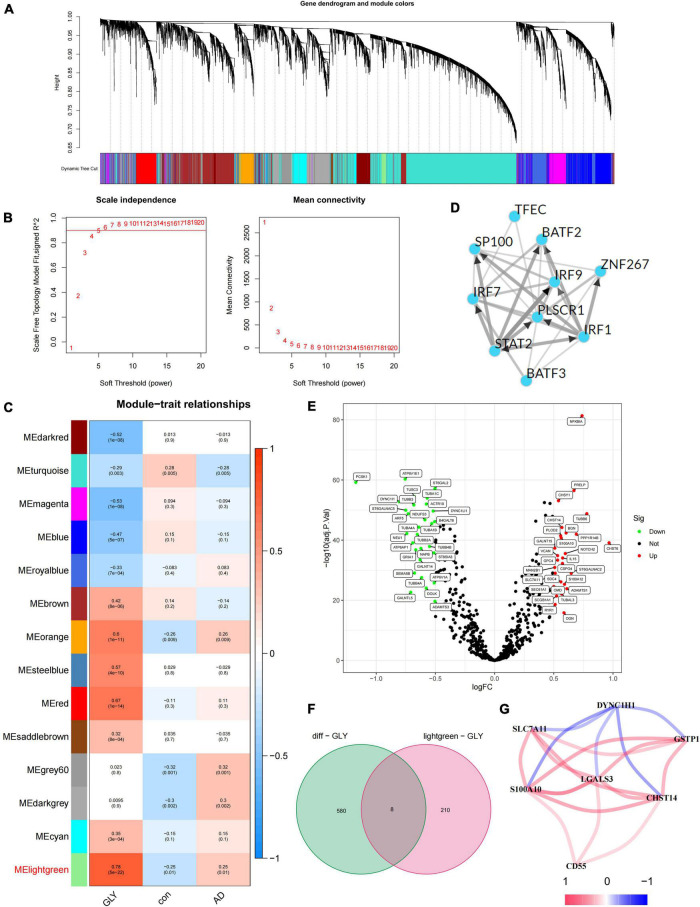
Identification of core protein glycosylation-related genes (PGRGs) in Alzheimer’s disease (AD). **(A)** Weighted gene co-expression network analysis (WGCNA) analysis was performed on the training set, resulting in a cluster dendrogram of co-expressed genes. **(B)** The soft threshold of a scale-free network. **(C)** The module-character relationship was constructed, with each module containing the corresponding correlation and *P*-value. **(D)** Transcription factors that regulate the expression of genes are represented by the MElightgreen module and their interactions. **(E)** Differentially expressed PGRGs between the training set and control samples. **(F)** The intersection of genes represented by the differentially expressed PGRGs and MElightgreen modules between the training set and control samples. **(G)** The co-expression relationship of 7 core PGRGs.

In this study, 672 PGRGs were involved, and the GLY of all samples was obtained using GSVA enrichment analysis. A scale-free network was constructed by combining the group information of the GLY and sample, and the soft threshold was set to 4 (Figures 1A,B). WGCNA identified 14 modules and assigned each a unique color (Figure 1C). By analyzing the correlation between phenotypes (AD or control samples) and GLY, the most associated module with AD and GLY was found to be the MElightgreen (Figure 1C), with a total of 218 genes. Genes in the same cluster often share common transcription factors; therefore, we predicted and analyzed the transcription factors of genes in the MElightgreen module and visualized the mutual regulatory relationships among the top 10 transcription factors in the Mean Rank (Figure 1D). The association of these transcription factors with asthma has been partially confirmed. For example, the *IRF7* rs6598008 polymorphism modulates the immune response to herpes simplex virus type 1 by affecting the IFN-λ pathway, which plays an important role in AD (Costa et al., 2017). There were 588 differentially expressed PGRGs in AD and control samples (Figure 1E), and eight core PGRGs were obtained from the de-intersection of genes in the MElightgreen module (Figure 1F). The co-expression relationship was explored using 0.2 as the critical value of the correlation coefficient, revealing that there was a close correlation among seven genes: *SLC7A11*, *S100A10*, *LGALS3*, *CD55*, *CHST14*, and *GSTP1* were positively correlated, while *DYNC1H1* was negatively correlated with the aforementioned genes (Figure 1G). These findings suggest that PGRGs may be associated with the development of AD.

### Core protein glycosylation-related genes divide Alzheimer’s disease into two subtypes

**FIGURE 2:**
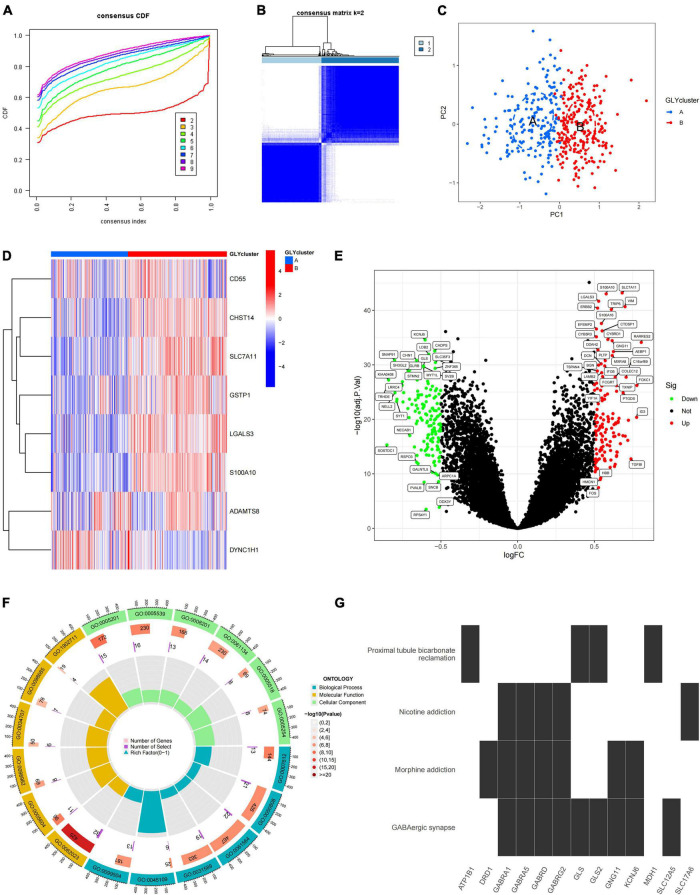
The two distinct protein glycosylation-related subtypes in Alzheimer’s disease (AD) identified by unsupervised clustering of eight protein glycosylation-related genes (PGRGs). **(A)** Consensus clustering cumulative distribution function (CDF) for *k* = 2–9. **(B)** Heatmap of the matrix of co-occurrence proportions of AD samples. **(C)** Principal component analysis (PCA) is used to determine the discrimination of A and B subtypes. **(D)** The difference in the expression of eight core PGRGs between the two subtypes. **(E)** Differentially expressed genes between the subtypes. **(F)** GO analysis of differentially expressed genes between the subtypes reveals related biological processes, molecular functions, and cellular components. **(G)** Kyoto Encyclopedia of Genes and Genomes (KEGG) enrichment analysis of differentially expressed genes between the subtypes.

**FIGURE 3:**
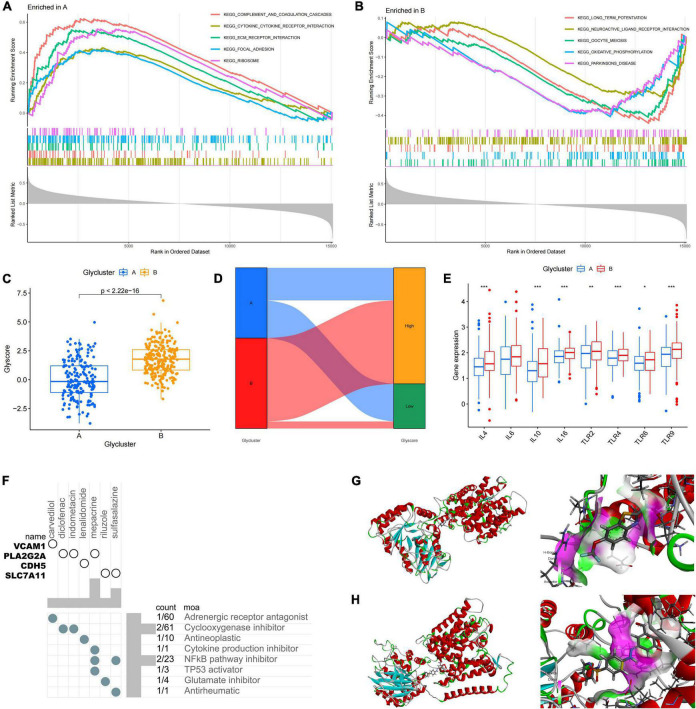
Differences in protein glycosylation levels, neuroinflammation, and drug prediction between the subtypes. **(A,B)** GSEA analysis of subtypes A and B. **(C)** The difference in glycosylation level between the subtypes. **(D)** The distribution proportion of protein glycosylation level in each subtype. **(E)** Differences in expression levels of cytokines and inflammatory signal transduction molecules between the subtypes. *P < 0.05, **P < 0.01, and ***P < 0.001. **(F)** The correlation between potential therapeutic drugs and corresponding targets. **(G)** The binding conformation of SLC7A11 and riluzole (binding energy = –7.8 kcal/mol). **(H)** The binding conformation of SLC7A11 and sulfasalazine (binding energy = –94.0 kcal/mol).

To study the role of PGRGs in AD, we conducted an unsupervised consensus cluster analysis on AD samples based on the expression of eight PGRGs. According to the cumulative distribution function curve and the heatmap of the matrix of co-occurrence proportions of AD samples, *k* = 2 was identified as the optimal number of clusters. PCA showed that there were significant differences in distribution between subtypes A and B, which indicated that there were two protein glycosylation-related molecular subtypes in AD (Figures 2A–C). The differences in the expression of the eight PGRGs between subtypes A and B are shown in Figure 2D. The analysis of expression differences revealed 273 DEGs between subtypes A and B (Figure 2E). To explore the biological functional differences between the subtypes, we performed GO and KEGG enrichment analyses of the DEGs and found that biological processes and molecular functions, such as axon development, vesicle-mediated transport in the synapse, associative learning, cognition, GABAergic synapse, GABA receptor complex, and inorganic anion transmembrane transporter activity, occupy the core position (Figures 2F,G). GSEA analysis showed that the signaling pathways of subtypes A and B were different (Figures 3A,B), and were mainly concentrated in the complement and coagulation cascades, cytokine–cytokine receptor interaction, long-term potentiation neuroactive ligand-receptor interaction, and others. After scoring the protein glycosylation levels of the two molecular subtypes, the Glyscore of subtype B was significantly higher than that of subtype A (Figure 3C), and most of the samples were identified as having a high Glyscore (Figure 3D). We compared inflammatory signaling molecules reported in AD between the subtypes to explore the association between protein glycosylation and neuroinflammation (Asby et al., 2021). The expression of inflammatory mediators or receptors, such as *IL4*, *IL6*, *IL10*, *IL16*, *TLR2*, *TLR4*, *TLR6*, and *TLR9*, was significantly upregulated in subtype B compared with those in subtype A (Figure 3E). These results suggest that high protein glycosylation levels are associated with more severe neuroinflammation and that core PGRGs are involved in the progression of AD and have a good classification function. To explore the differences in potential therapeutic drugs in patients with different glycosylation levels, DEGs between subtypes A and B were entered into the cMAP database, and seven small molecule drugs targeting protein glycosylation levels were screened. These drugs were mainly adrenergic receptor antagonists, cyclooxygenase inhibitors, glutamate inhibitors, and NF-κB pathway inhibitors that were significantly correlated with VCAM1, PLA2G2A, CDH5, SLC7A11, and other targets (Figure 3F). To confirm their binding ability, the previously studied drugs riluzole and sulfasalazine were selected for the analysis of molecular docking with SLC7A11. The results showed that the affinity between them was less than −5 kcal/mol. The molecular docking model is shown in Figures 3G,H.

### Diagnostic nomogram model construction, assessment, and diagnostic marker screening between the two subtypes

**FIGURE 4:**
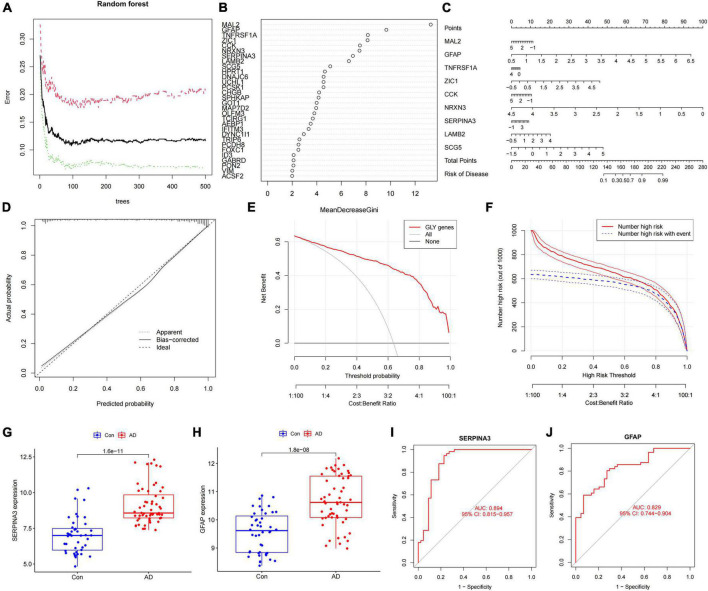
Construction and verification of the diagnostic line diagram model **(A)** Random forest trees constructed by cross-validation. **(B)** Genes with an importance score higher than 2. **(C)** A line chart was used to predict different protein glycosylation levels in patients with Alzheimer’s disease (AD). **(D)** A calibration curve that evaluates the predictive ability of the line chart model. **(E)** The decision curve analysis (DCA) curve was used to evaluate the clinical value of the line chart model. **(F)** The clinical impact curve of the line chart model constructed based on the DCA curve. **(G)** The difference in the *SERPINA3* gene expression between AD and control samples. **(H)** The difference in the *GFAP* gene expression between AD and control samples. **(I)** Receiver operating characteristic curve of the *SERPINA3* gene in the validation set. **(J)** Receiver operating characteristic curve of the *GFAP* gene in the validation set.

Differentially expressed genes between subtypes A and B were screened by constructing random forest trees, and genes with importance scores greater than 2 were sequenced (Figures 4A,B). A diagnostic line diagram model was established based on the genes with importance scores greater than 5 (*MAL2*, *GFAP*, *TNFRSF1A*, *ZIC1*, *CCK*, *NRXN3*, *SERPINA3*, *LAMB2*, and *SCG5*) (Figure 4C). Calibration curves showed that the error between the actual and predicted risks was very small, confirming the high accuracy of the line diagram model in predicting glycosylated protein molecular subtypes (Figure 4D). DCA showed that the “GLY genes” curve was higher than the gray curve, indicating that patients could benefit clinically within the high-risk threshold range from 0 to 1 (Figure 4E). To evaluate the clinical effect of the rosette model more directly, a clinical effect curve was constructed based on the DCA curve. When the high-risk threshold ranges from 0.4 to 1, the curve of “Number of High Risks” is close to that of “Number of High Risks with Events,” indicating that the line diagram model has a good prediction ability (Figure 4F). To test the expression level and diagnostic value of the genes contained in these line diagram models, external validation of nine genes was carried out using the GSE122063 (Figures 4G–J), GSE118553 (Supplementary Figures 1A–D), GSE44768 (Supplementary Figures 1E–H), and GSE44770 (Supplementary Figures 1I–L) datasets, and the results showed that the difference trend of SERPINA3 and GFAP was relatively stable, and their expression was upregulated in AD samples. These results suggest that *SERPINA3* and *GFAP* may play key roles in the pathological progression of AD mediated by protein glycosylation at different levels.

### Immune cell infiltration between the subtypes and its correlation with protein glycosylation-related genes

**FIGURE 5:**
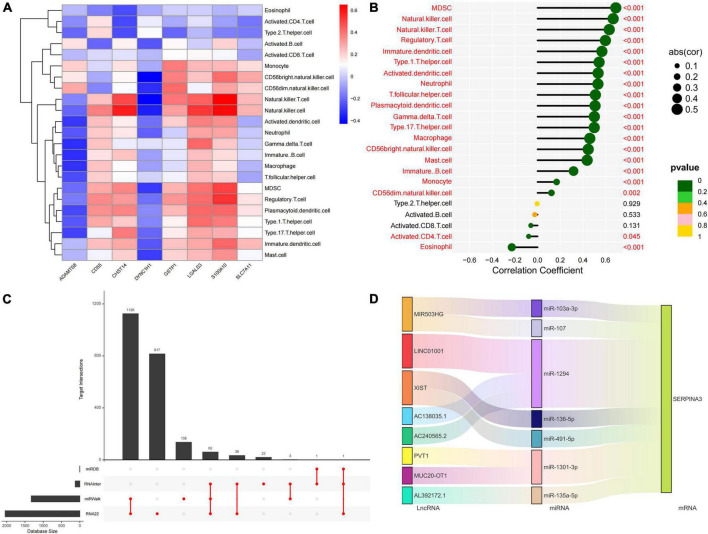
Immune cell infiltration profiles across the key diagnostic marker subtypes and expression regulatory networks. **(A)** The correlation between the expression of eight core PGRGs and immune cell infiltration. **(B)** The correlation between the *SERPINA3* gene and immune cells. **(C)** The intersection of miRNAs by which the expression of *SERPINA3* is regulated in four databases. **(D)** The regulatory ceRNA network of *SERPINA3* expression.

We quantified the level of immune cell infiltration to assess the immune landscapes of subtypes A and B. The results showed significant differences in immune cells, except for activated CD8^+^ T cells and macrophages. Interestingly, type 2 immunity, represented by eosinophils and type 2 T helper cells, was significantly downregulated in subtype B, whereas Th1, Th17, and B cells were significantly upregulated, indicating abundant immunocyte heterogeneity in brain tissue under the influence of protein glycosylation (Supplementary Figure 1M). At the same time, there was a strong–generally positive–correlation among immune cells (Supplementary Figure 1N). In addition, among the eight core PGRGs, the expression of *S100A10* and *LGALS3* were positively correlated with the level of immune cell infiltration, whereas that of *DYNC1H1* was negatively correlated (Figure 5A). Spearman correlation analysis showed that *SERPINA3*, the diagnostic marker with the highest area under the receiver operating characteristic curve (AUC) and the most significant difference between the subtypes, was significantly correlated with all immune cells, except type 2 T helper cells, activated B cells, and activated CD8^+^ T cells (Figure 5B). Finally, to determine the gene expression regulatory network of *SERPINA3*, we explored its ceRNA mechanism and obtained 63 miRNAs that may regulate its expression level, of which seven were identified after literature screening. Finally, a ceRNA network, including eight lncRNAs and seven miRNAs, was constructed (Figures 5C,D).

## Discussion

Protein glycosylation has been widely studied in AD because of its important role in regulating the biological functions of the brain (Schedin-Weiss et al., 2014; Ansari and Emerald, 2019; Haukedal and Freude, 2020). Recent reports have shown that glycosylation levels in the frontal cortex of patients with AD are increased and that N-linked glycan dysregulation could underpin AD pathologies (Hawkinson et al., 2021). This may be related to complex immune cell regulation, such as abnormal glycosylation of microglia, which can affect neuroinflammation and mediate AD progression. Accordingly, some reports have discussed the possibility of using glycans or glycoproteins as biomarkers of AD (Palmigiano et al., 2016). In this study, we identified eight central genes that may be involved in protein glycosylation-related pathological processes in AD, and the relationship between most of these genes and AD was studied.

*CD55* is a regulatory factor for C3 and C5 invertases and regulates the complement system by binding with C3b and C4b to protect neurons from the influence of the complement system (Wang et al., 2010; Helgadottir et al., 2019). *CD55* is also involved in neuroinflammation in cooperation with CD97 to promote B- and T-cell proliferation, both of which play important roles in the pathogenesis of AD (Cao and Zheng, 2018). Studies have reported that these two regulatory mechanisms of CD55 are related to protein glycosylation, and the abnormal glycosylation of the CD55 protein can cause a severe loss of its regulatory activity, leading to the upregulation of complement system activity, increased tissue damage, and abnormal T-cell activation (Flückiger et al., 2018). S100A10 is a specific marker of A2 astrocytes, which is a subtype of astrocytes with neuroprotective effects that upregulates many neurotrophic factors (Liddelow et al., 2017; King et al., 2020). The neurotoxicity of Aβ is an important cause of synaptic damage and neuronal death in the brains of patients with AD. LGALS3 mediates Aβ deposition by regulating the activation of microglia and induces neuroinflammation and cognitive impairment in AD, and therefore is a key participant in AD pathophysiology (Sethi et al., 2021; Tan et al., 2021). LGALS3 inhibitors have been widely developed and synthesized. In addition, compared with normal brain tissue, LGALS3 protein has significant N-glycosylation at amino acids 192 and 398, but the biological significance of this modification is not yet understood (Zhang et al., 2020). The complex roles of these PGRGs in AD indicate the potential value of protein glycosylation-related targets at the drug development level.

Neuroinflammation is generally considered an important part of the pathological changes in the AD brain (Serrano-Pozo et al., 2021). Our results suggest that there are differences in the interactions between cytokines and receptors among different protein glycosylation level subtypes. Clinical studies have found that serum IL-4, IL-6, IL-10, and IL-16 levels in patients with AD are significantly higher than those in healthy individuals, and some inflammatory factors are correlated with cognitive function, language, and memory in patients with AD (Motta et al., 2007; Lu et al., 2022). During inflammation, the expression of the interleukin family proteins is upregulated in the central nervous system, which contributes to the pathological processes of AD. A mouse model of astrocyte-targeted IL-6 production showed significant neuronal overexcitation and progressive cognitive decline, and the pathological changes in the central nervous system included neurodegeneration, demyelination, and microglial proliferation (Campbell et al., 1993; Steffensen et al., 1994; Heyser et al., 1997). Knocking out *IL10* can promote the phenotypic transformation of microglia and enhance their phagocytosis of Aβ oligomers, which may be due to the negative regulation of microglial phagocytosis through the IL-10R/STAT3 pathway (Liu et al., 2021). The phagocytosis of Aβ by microglia is also TLR2-dependent, and Aβ can be used as an agonist of microglial TLR2 (Schütze et al., 2012). Another TLR family member, TLR4, induces a pro-inflammatory response to Aβ by stimulating the activation of microglia, uptake and clearance of Aβ, and neuronal apoptosis (Adhikarla et al., 2021). Interestingly, among the drugs predicted to have potential clinical applications, riluzole was also shown to be strongly associated with neuroinflammation and protein glycosylation. Fluctuations in neuronal glucose levels can cause dramatic changes in protein glycosylation levels, and a double-blind, randomized, placebo-controlled study showed that riluzole slowed the rate of decline in cognitive and cerebral glucose metabolism in patients with AD. This effect may be related to riluzole’s role in activating the Wnt/β-catenin pathway, inhibited in AD, which is sensitive to changes in cellular glycosylation status and mediates the occurrence of neuroinflammation (Bukke et al., 2020; Vallée et al., 2020; Matthews et al., 2021). More importantly, glutamate hyperactivation of the N-methyl-D-aspartate (NMDA) receptor is positively associated with neurodegenerative disease, and the basic pharmacological effect of riluzole is to produce neuroprotective and antidepressant effects by reducing glutamate release. This was consistent with the inclusion of glutamate inhibitor in the drug prediction results (Reisberg et al., 2003). Another drug, sulfasalazine, also attenuates neuroinflammation, possibly by inhibiting the NF-κB signaling pathway (Dijkstra et al., 2002; Ma et al., 2018). This class of NF-κB signaling pathway inhibitors function by downregulating the activity of NF-κB, inhibiting the expression of neurotoxic cytokines and chemokines, and attenuating Aβ production to block the progression of AD (Sivandzade et al., 2019). The other two drugs in the drug prediction results were also associated with neuroinflammation. Among them, selective COX-1 inhibitors changed the phenotype of microglia, reduced neuroinflammation, and reduced the deposition of amyloid and tau proteins (Choi et al., 2013). The mechanism of adrenergic receptor antagonists are more complex. In addition to coordinating with the above cyclooxygenase inhibitor to block the release of inflammatory mediators, they also improve cognitive function in AD patients by inhibiting the matrix metalloproteinase and mitogen-activated protein kinase pathways (Lu’o’ng and Nguyen, 2013). In addition to cytokines and inflammatory signal transduction molecules, plasma from patients with AD showed changes in specific immunoglobulin glycosylation, providing more evidence for the correlation between neuroinflammation and glycosylation in AD (Lundström et al., 2014). However, the exact mechanism still needs to be further analyzed with the support of extensive data of the glycoprotein groups, single-cell transcriptome, and spatial transcriptome.

As an immune cell type, the role of microglia in AD has been recognized, but other cells that are involved in the innate immune system are also affected by polysaccharide modification. Although the delivery of immune cells from the peripheral circulation to the central nervous system is tightly regulated, the blood–brain barrier and meningeal lymphoid function are impaired in AD, resulting in immune cell transport changes. Studies have shown that there are a small number of adaptive immune cells in the brain parenchyma, such as CD4^+^ T and CD8^+^ T cells, and their infiltration increases with age (Mrdjen et al., 2018; Pasciuto et al., 2020). In fact, in AD, the infiltration of T cells into the central nervous system results in a decrease in the ability of microglia to clear Aβ (Ferretti et al., 2016). It is worth noting that there are many kinds of differentiated and mature T cells in the central nervous system (Browne et al., 2013). Activated Th17 cells can produce pathogenic IL-17A, which enhances the inflammatory cascade response by recruiting neutrophils and promoting neuroinflammation and neurodegeneration in AD. Th17 cells can also interact with other immune cells, together contributing to AD pathology. In this study, we found a positive correlation between Th17 cells and macrophages, in line with previous reports, indicating that microglia exposure to IL-17A leads to the activation and production of pro-inflammatory cytokines, resulting in more severe neuronal dysfunction (Sun et al., 2015). These results suggest that immune cells are important contributors to neuroinflammation in AD; however, current studies have only confirmed the potential relationship between microglia and glycosylation in the progression of neuroinflammation in AD. The results of our study may have implications on the relationship between other immune cells and protein glycosylation in AD for further studies.

There are some limitations to our study. First, the clinical information contained in the dataset is limited, and important information such as the history of drug use and the severity of symptoms cannot be obtained, which may bring potential bias to the data analysis. Moreover, this study did not combine protein glycosylation to analyze the clinical characteristics of AD patients. Second, the line chart model may need to be further tested before clinical application because there is no prognostic information or survival time in the current version of the model. Finally, the expression levels of the diagnostic marker genes involved in the line diagram model need to be verified by further experiments.

## Conclusion

In this study, we identified eight central genes (*SLC7A11*, *S100A10*, *LGALS3*, *CD55*, *CHST14*, *GSTP1*, *DYNC1H1*, and *ADAMTS8*) closely related to protein glycosylation in AD, which can classify AD patients into two subtypes. There was strong heterogeneity in the level of immune cell infiltration between the subtypes, and SERPINA3 was closely related to immune cells as a diagnostic marker to discriminate between the subtypes. Studies have found that riluzole and sulfasalazine have potential value in treating patients with high protein glycosylation and have preliminarily established the relationship between protein glycosylation in AD and neuroinflammation. However, further research is required to confirm the clinical value of our findings.

## Data availability statement

The datasets presented in this study can be found in online repositories. The names of the repository/repositories and accession number(s) can be found in the article/Supplementary material.

## Author contributions

ZM and XR designed and conducted the whole research. TM and XL carried out molecular biological analysis. QW and HS applied for the GEO dataset analysis of Alzheimer. FY, WC, and YL completed the data analysis and drafted the manuscript. JF, XR, and YM revised and finalized the manuscript. All authors contributed to the article and approved the submitted version.

## Footnotes

1
https://www.ncbi.nlm.nih.gov/geo/

2
https://acgg.asia/ggdb2/index

3
https://www.genenames.org/data/genegroup/#!/group/424

4
https://www.genecards.org/

5
https://maayanlab.cloud/chea3/

6
https://pubchem.ncbi.nlm.nih.gov/

7
https://clue.io/command?q=/home

8
https://www.rcsb.org/

9
www.mirnet.ca/miRNet/home.xhtml

10
http://starbase.sysu.edu.cn/
