# Supplementary figures and images for: Identification and immune characteristics of molecular subtypes related to protein glycosylation in Alzheimer’s disease
Source: Front Aging Neurosci. 2022 Nov 2;14:968190. doi: 10.3389/fnagi.2022.968190 (PMC9667030; doi:10.3389/fnagi.2022.968190)

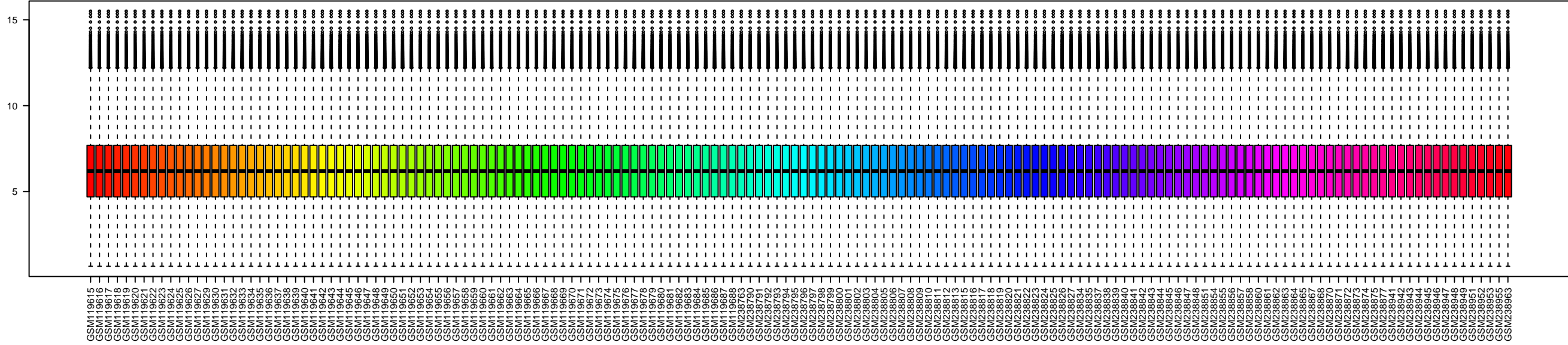

Supplement: Supplementary file 1 [file Data_Sheet_1.PDF]

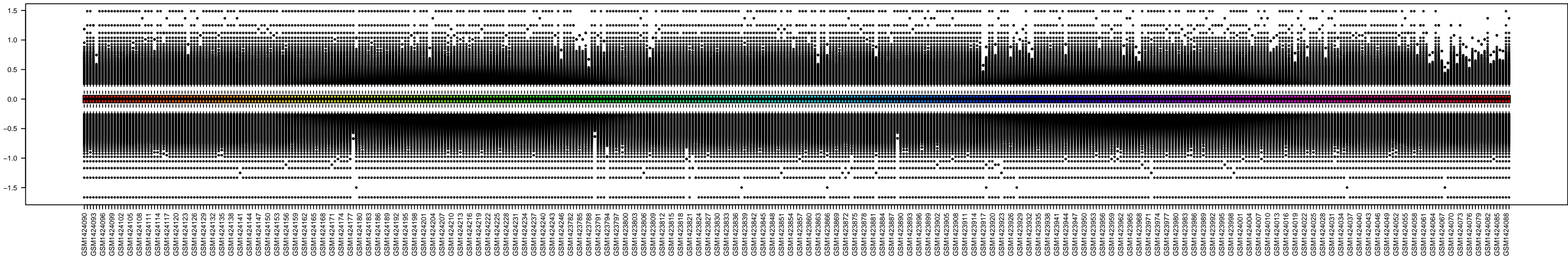

Supplement: Supplementary file 2 [file Data_Sheet_2.PDF]

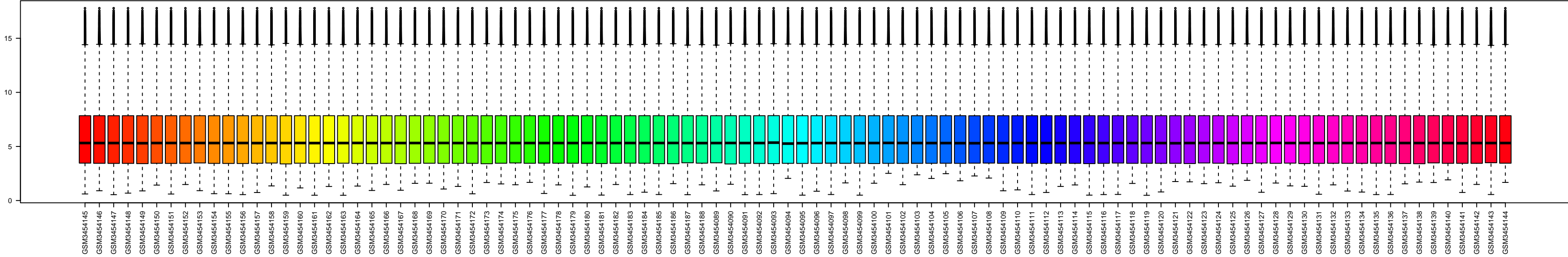

Supplement: Supplementary file 3 [file Data_Sheet_3.PDF]

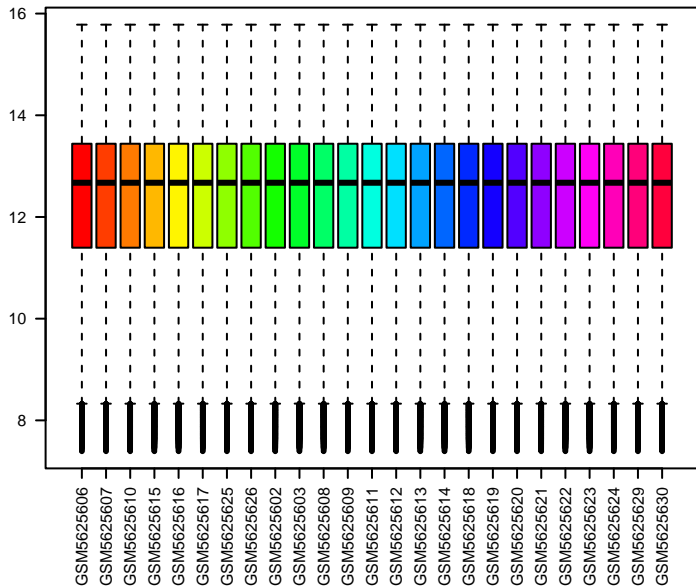

Supplement: Supplementary file 4 [file Data_Sheet_4.PDF]
