# Supplementary material for: Identification and immune characteristics of molecular subtypes related to protein glycosylation in Alzheimer’s disease
Source: Front Aging Neurosci. 2022 Nov 2;14:968190. doi: 10.3389/fnagi.2022.968190 (PMC9667030; doi:10.3389/fnagi.2022.968190)
Supplement: Supplementary file 5 [file Image_1.pdf]

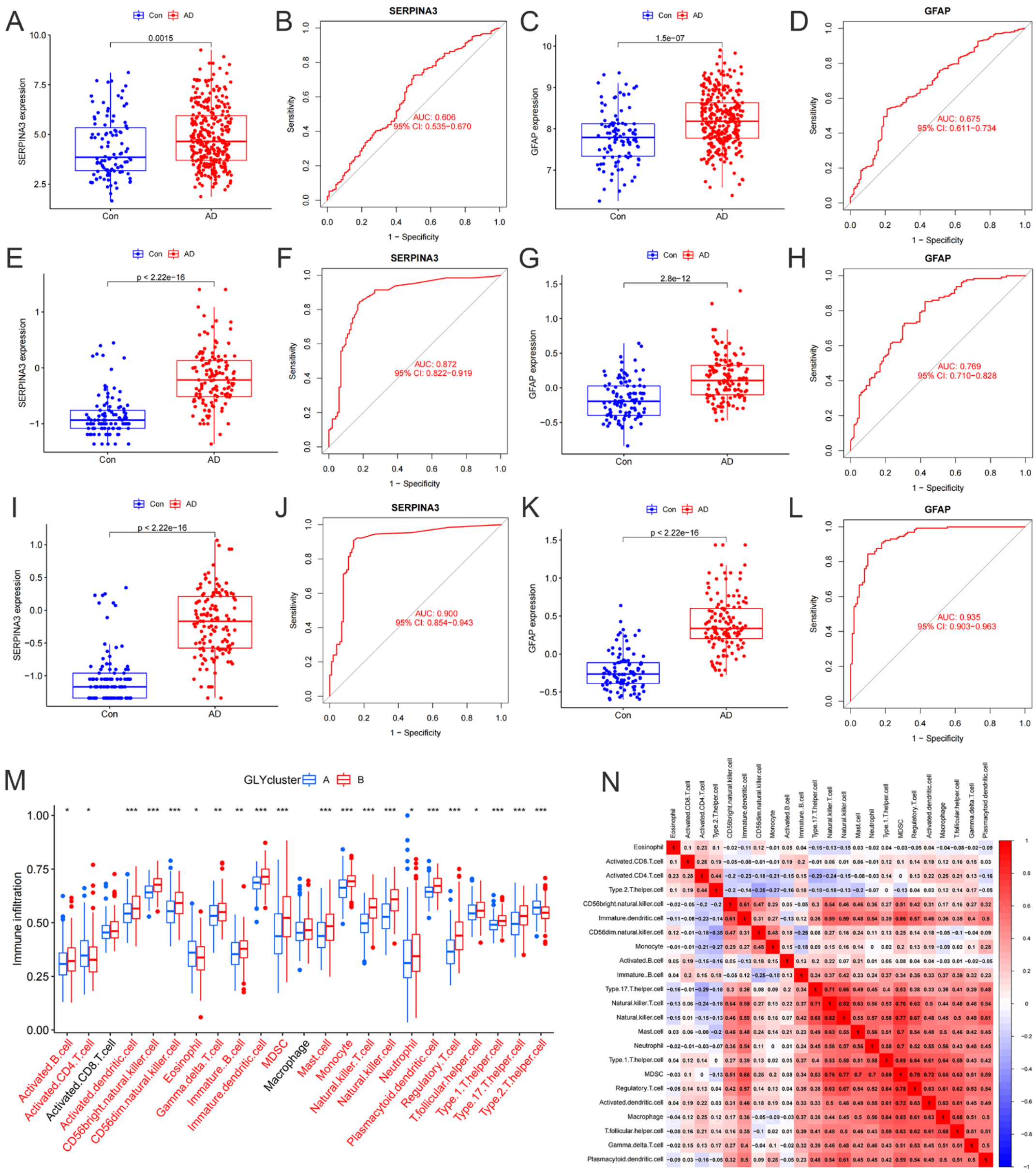

Figure S1 (A) The difference in the SERPINA3 gene expression between AD and control samples in GSE118553 dataset. (B) The difference in the GFAP gene expression between AD and control samples in GSE118553 dataset. (C) Receiver operating characteristic curve of the SERPINA3 gene in GSE118553 dataset. (D) Receiver operating characteristic curve of the GFAP gene in GSE118553 dataset. (E) The difference in the SERPINA3 gene expression between AD and control samples in GSE44768 dataset. (F) The difference in the GFAP gene expression between AD and control samples in GSE44768 dataset. (G) Receiver operating characteristic curve of the SERPINA3 gene in GSE44768 dataset. (H) Receiver operating characteristic

curve of the GFAP gene in GSE44768 dataset. (I) The difference in the SERPINA3 gene expression between AD and control samples in GSE44770 dataset. (J) The difference in the GFAP gene expression between AD and control samples in GSE44770 dataset. (K) Receiver operating characteristic curve of the SERPINA3 gene in GSE44770 dataset. (L) Receiver operating characteristic curve of the GFAP gene in GSE44770 dataset. (M) The difference in the immune cell infiltration between the subtypes. (N) Correlation heatmap of immune cells, with values of squares as correlations. The red color indicates a positive correlation, and the blue color indicates a negative correlation.
